# Moisture-Dependent Physical-Mechanical Properties of Maize, Rice, and Soybeans as Related to Handling and Processing

**DOI:** 10.3390/ma15248729

**Published:** 2022-12-07

**Authors:** Weronika Kruszelnicka, Zhengpu Chen, Kingsly Ambrose

**Affiliations:** 1 Department of Renewable Energy Sources Engineering and Technical Systems, Faculty of Mechanical Engineering, Bydgoszcz University of Science and Technology, Al. Prof. S. Kaliskiego 7, 85-796 Bydgoszcz, Poland; 2 Agricultural and Biological Engineering, Purdue University, 610 Purdue Mall, West Lafayette, IN 47907, USA

**Keywords:** grain kernels, moisture content, strength, compression test, size, shape, breakage energy, sphericity

## Abstract

Knowledge of physical and mechanical properties of cereal grains is important for designing handling and processing equipment. However, there is still a lack of knowledge on the influence of moisture content on the physical-mechanical properties as related to machine design. The aim of this study was to investigate and describe the changes in select physical-mechanical properties of maize, rice, and soybeans at various moisture content (10%, 14%, 18%, 22%, 26%; wet basis) and their compression behavior at two loading rates of 1.25 mm/min and 125 mm/min. The measured physical and mechanical properties include size, shape, and breakage force of single kernels. It was found that an increase in moisture content increased the kernel size, altered the kernel shape, and decreased the bulk density. The effects of moisture content and loading rate on breakage force, stress, and energy varied depending on the grain type. Our results indicated that an increase in moisture content changed the mechanical behavior of grain kernels from brittle to viscoelastic. To prevent kernel damage during processing and handling, the measured force and stress during compression can be used as the limit value for designing equipment.

## 1. Introduction

The physical and mechanical properties of cereal grains play an important role when designing machines for grinding, conveying, extrusion, compaction, etc. [1,2,3,4]. For example, it is important to know the bulk density when designing storage containers, as it influences the load and pressure distribution [5]. The size and shape of cereal grains influence the design of planting, harvesting, and separation equipment [1]. In addition, changes in grain size result in variation in cohesive strength and wall friction angle, impacting the flowability, which is the one of the parameters that defines the optimal angle for hoppers [6], conveyors [7], feeders, and outlet dimensions in storage containers [8]. The shape of grains and their surface area affect the moisture diffusivity, impacting the drying behavior and aeration during storage [1]. Furthermore, size and shape are also important input parameters for discrete element method (DEM) simulation of bulk grain [9,10]. The prediction accuracy of the DEM models depends on the type of grains and contact parameters, which are related to the kernel size and shape [11]. 

It is well known that the variability in physical properties of cereal grains is dependent on the moisture content (MC) [1,11,12]. In particular, with fluctuation in relative humidity and temperature, the moisture content of cereal grains also changes [11,13]. Moreover, MC is adjusted based on the processing condition requirements. For long-term storage, the grains are dried to low MC levels to prevent mold growth, while higher MC levels are preferred during harvesting to reduce grain damage [14,15]. 

Rice, maize, and soybeans are the most commonly processed grains by the food and feed industry. Rice, in the form of raw and milled grains, flour, noodles, etc., is the source of food for almost one third of the world population [16]. Maize is the highest produced cereal grain worldwide [17] and is processed for starch, oil, and flour, in addition to whole grains consumption. Meanwhile, cultivation of soybean is growing every year. In the years 2021–2022 the cultivation area of soybean was about 130.94 million ha worldwide and total annual production was estimated at 355.59 MMT [18]. These cereal grains are subjected to various processing using a wide range of mechanical devices, and knowledge about changes in their physical and mechanical properties will help improve the processing and the quality of the final product [19].

Rice, soybean, and corn grains are processed at a variety of moisture content levels depending on the processing type [14,15]. Different moisture contents are required, for example, for storage (preferably low to reduce the risk of mold development, for rice as low as 9% MC [14], for corn up to 14% MC [20]) and for harvesting (for medium rice about 22–24% MC [15], for corn below 28% MC [21]), and grains with different MC will be suitable, for instance, for comminution or milling (for rice 12–14% MC [14], for corn 14–15% MC [20]). The physical and mechanical properties in this study were analyzed for grains with the MC ranged from 10% to 26%, to cover the MC ranges of grains processing.

Many published studies have reported the influence of MC on physical properties such as size, angle of repose, and static friction for rice [22], maize [23], and soybeans [24]. However, very little attention is paid to the parameters describing their mechanical behavior such as forces, strength, deformation, and breakage energy. Grain damage due to breakage is common during harvesting and handling, which decreases the grain quality. On the other hand, cereal grains are subjected to processing operations where breakage is needed, e.g., starch extraction, ethanol production, etc. [23]. Published studies have shown that the moisture content, genotype, and chemical composition are the factors that impact the mechanical behavior and breakage susceptibility of grain kernels [12,25,26]. For example, maize kernels with corneous endosperm are more resistant to high loads, and their hardness is higher than maize with soft endosperm [27]. In addition, an increase in moisture content results in kernel softening and ductility [12,28]. Other factors affecting the behavior of kernels subjected to external loads are the size, shape, loading rate, and grain orientation [19,29,30]. A higher loading rate can result in an increase or decrease in force, deformation, and breakage energy depending on the type of grain and its internal structure [28,30,31]. The differences in mechanical properties are directly correlated with grinding and milling performance indicators such as energy consumption and efficiency [3,32]. 

The aim of this research was to investigate and describe the changes in important physical-mechanical properties of maize, rice, and soybean grains at different moisture content levels. The effect of moisture content on the size, shape, and mechanical characteristics such as breakage force, deformation, and stress were studied at five levels of moisture content, 10%, 14%, 18%, 22%, and 26% (wet basis, w.b.). The measurements included three characteristic dimensions, the weight of kernels, and force-deformation characteristics from a single grain kernel compression test.

## 2. Materials and Methods

### 2.1. Sample Preparation

Maize, soybeans, and rice were chosen for this study because they are the most processed grains by the food and feed industry. The maize and soybeans were acquired from Purdue University research farm in West Lafayette, IN, USA. The rice grains, a medium grain variety white rice (NISHIKI Brand), was purchased from a local store. Five different levels of moisture content, 10%, 14%, 18%, 22%, and 26% (w.b.), at the range in which these grains are processed were used in this study. A rewetting procedure was conducted to obtain the samples at the predetermined moisture content level. A total of 8 kg of samples were rewetted for each grain type. First, the initial moisture content of grains was determined using the standard air oven technique. Then, the calculated amount of water was added to increase the moisture content of the samples to 28% (w.b.). The mass of water needed for rewetting was calculated using Equation (1) [1]:

(1) $$Q=\frac{MC_{2}-MC_{1}}{100-MC_{2}}\cdot m$$

where *Q* is the amount of water (L), *m* is the weight of the grain at the initial moisture content (kg), *MC*_2_ is the desired moisture content of the grain (%), and *MC*_1_ is the initial moisture content of the grain (%).

In order to obtain uniform rewetting, the grain samples were mixed in a rotating drum for 24 h and stored for 72 h at 5 °C. After determining the moisture content of rewetted samples, the grains were then divided into 5 smaller subsamples using the Boerner divider. To obtain the samples at desired moisture content, the subsamples were spread in a tray and dried at room temperature.

### 2.2. Moisture Content Measurement

For maize and soybeans, the MC was determined following the American Society of Agricultural and Biological Engineers (ASAE) S352.2 standard [33], which recommends drying the grains at the temperature of 103 °C for 72 h in a hot air convection oven. Rice kernels were dried at a temperature of 130 °C for 16 h in a hot air convection oven per International Rice Research Institute (IRRI) guidelines [34]. The MC measurement was done in 3 replications involving 15 g samples (100 g was used in the case of maize after rewetting, as recommended by the ASAE S352.2 standard for grains with MC higher than 25%). The mass of samples before and after drying was recorded with an accuracy of 0.001 using the Mettler Toledo ME203E analytical scale, and the MC was determined from the loss in weight and reported in wet basis. 

### 2.3. Bulk Density Characterization

The bulk density (*ρ_B_*) was measured with the one-pint test weight apparatus, according to the standard test weight procedure [35]. Bulk density was determined for grains at the initial MC and after rewetting. For each MC level, three replicates were done. The bulk density was calculated from the mass and volume using Equation (2):

(2) $$\rho_{B}=\frac{M}{V}$$

where *M* is the sample mass (g) and *V* is the container volume, which equals 550.6 cm^3^.

### 2.4. Size Measurement

**Figure 1:**
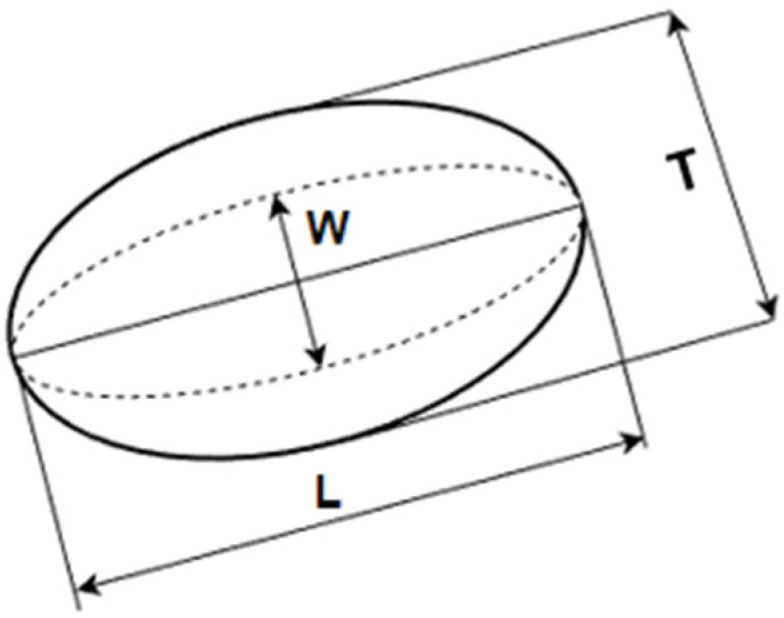
Representation of dimensions measured for each grain kernel: width (W), length (L), and thickness (T).

The size measurement was done for a representative sample of 100 kernels of rice, maize, and soybeans at each MC level. For each grain kernel, the width (W), length (L), and thickness (T) (Figure 1) were measured with a digital caliper. 

From the three dimensions, the equivalent diameter *D_e_* of each kernel was calculated using Equation (3) [36]:

(3) $$D_{e}={\left(\frac{L\cdot {\left(W+T\right)}^{2}}{4}\right)}^{\frac{1}{3}}$$

The weight of each kernel was measured using the ME203E analytical scale (Mettler Toledo, Columbus, OH, USA) with an accuracy of 0.001 g.

### 2.5. Shape Determination

The indices chosen to describe the shape of the kernels include the sphericity index (*f*), aspect ratio (*AR*), and flatness ratio (*FR*), and were calculated using the formula given below [19,36]:

(4) $$f=\frac{{\left(L\cdot W\cdot T\right)}^{\frac{1}{3}}}{L}$$

(5) $$AR=\frac{W}{L}$$

(6) $$FR=\frac{T}{W}$$

### 2.6. Compression Test

**Figure 2:**
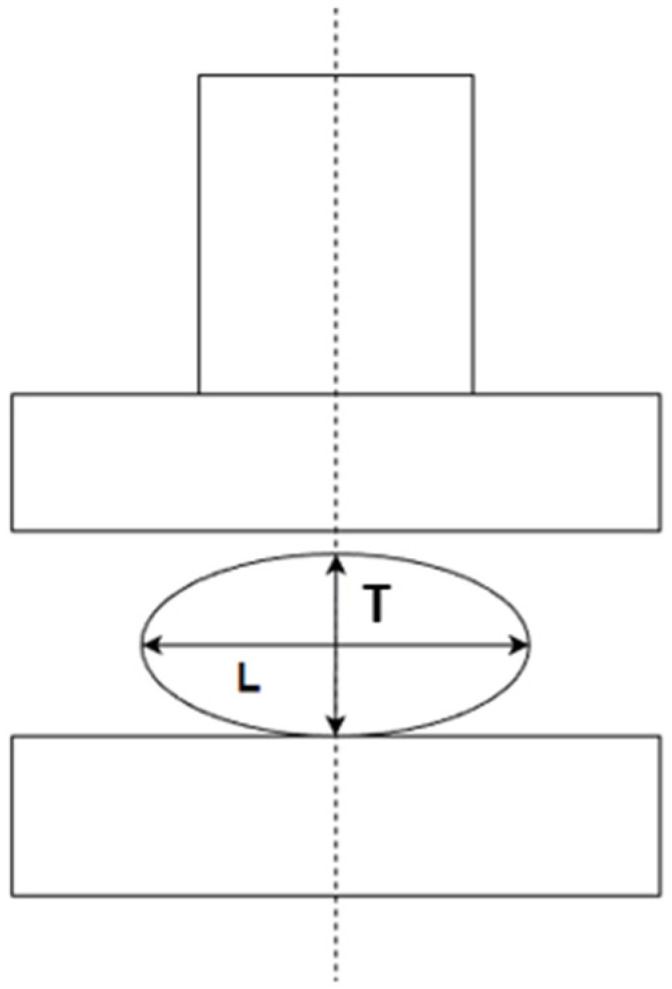
Grain position between parallel plates: length (L), thickness (T).

The compression test was performed at two loading rates, 1.25 mm/min and 125 mm/min, for 100 grain kernels to determine the breakage force, deformation, and strength at low and high velocities of loading. The compression tests were conducted using the MTS testing machine (MTS Systems Corporation, 14000 Technology Drive, Eden Prairie, MN, USA) with the 5 kN load cell (force measurement accuracy of ±0.001 N and deformation measurement accuracy of 0.001 mm). During compression testing, a single kernel was placed between the two parallel plates in a horizontal position (Figure 2), which is the natural position that prevents grain movement during loading [37]. The test was stopped at the first break, and the force and deformation at this point (also known as the bioyield point) were recorded. In this study, deformation was defined as the change in the grain dimension, which equals the load head displacement. The load cell and plate deformation during loading were neglected. 

The strength of the particles was calculated according to Equation (7) [36,38]:

(7) $$\sigma =\frac{F_{B}}{A_{P}}=\frac{F_{B}}{\frac{\pi LW}{4}}$$

where *F_B_* is the force inducing the rupture (N), and *A_p_* is the area of the compressed sample (mm^2^).

The energy needed for the breakage initiation was calculated from the area under the force–displacement curve [30].

### 2.7. Statistical Analysis

The data were subjected to statistical analysis after removing up to 20% outliers (data with values more than two standard deviations from the mean). The descriptive statistics including mean, median, and standard deviation were performed for each data set. The Pearson correlation analysis and regression analysis were conducted to identify the relationships between MC and the physical and mechanical properties at a significance level of 0.05. The null hypothesis was set that the moisture content is not responsible for changes in selected physical-mechanical properties. The null hypothesis is rejected when the *p*-value (probability) is lower than the adopted significance level (0.05). The rejection of the null hypothesis means that the changes in the selected physical-mechanical properties are caused by the moisture content changes rather than a random event. The F-test (Fisher test) was performed to choose between linear and polynomial regression models. The null hypothesis in this test was set that model 2 (quadratic) does not fit the data significantly better than model 1 (linear). The null hypothesis is rejected when the value of F is greater than the critical value of the F-distribution with a false rejection probability of 0.05. A two-way ANOVA was performed to check the significant differences in means between breakage forces, stresses, deformations, and energies at both loading rates. For pairwise comparisons, the post hoc Fisher least significant difference (LSD) test was done at a 95% confidence level.

## 3. Results and Discussion

### 3.1. Effect of Moisture Content on the Size of Grain Kernel

Table 1 presents the values of exact moisture content at five selected levels after drying for rice, maize, and soybeans. For simplicity, throughout the discussion, the moisture content values are rounded off to the nearest whole number. 

**Figure 3:**
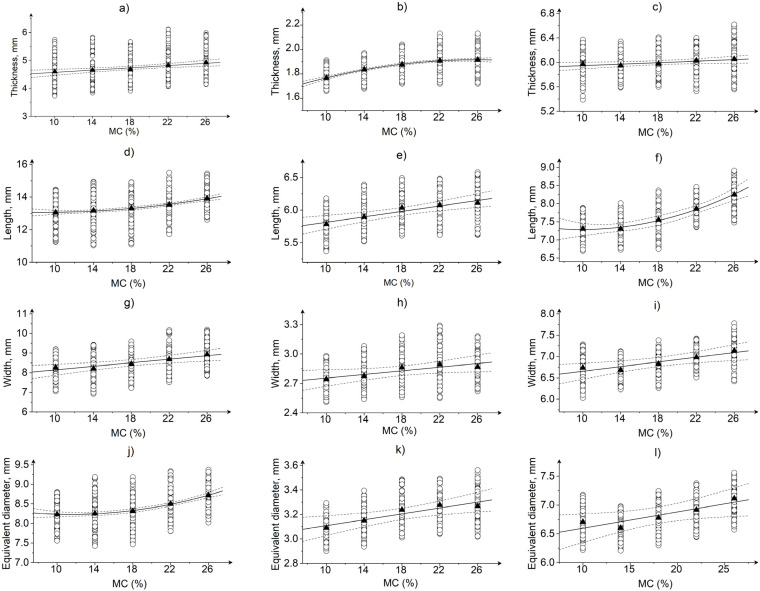
The dependencies between moisture content and thickness: (**a**) maize, *T* = 0.0205 *MC* + 4.438, R^2^ = 0.923, (**b**) rice, *T* = −5.95 × 10^−4^ *MC*^2^ + 0.0306 *MC* + 1.522, R^2^ = 0.999, (**c**) soybeans, *T* = 0.00602 *MC* + 5.887, R^2^ = 0.786; length: (**d**) maize, *L* = 0.00272 *MC*^2^ − 0.0463 *MC* + 13.253, R^2^ = 0.996, (**e**) rice, *L* = 0.0209 *MC* + 5.605, R^2^ = 0.928, (**f**) soybeans, *L* = 0.00374 *MC*^2^ − 0.0736 *MC* + 7.747, R^2^ = 0.994; width: (**g**) maize, *W* = 0.0448 *MC* + 7.700, R^2^ = 0.895, (**h**) rice, *W* = 0.0925 *MC* + 2.663, R^2^ = 0.796, (**i**) soybean, *W* = 0.0273 *MC* + 6.382, R^2^ = 0.874; equivalent diameter: (**j**) maize, *D_e_* = 0.00233 *MC*^2^ − 0.0534 *MC* + 8.536, R^2^ = 0.997, (**k**) rice, *D_e_* = 0.0120 *MC* + 2.988, R^2^ = 0.877, (**l**) soybeans, *D_e_* = 0.00283 *MC* + 6.314, R^2^ = 0.808; ○—data, ▲—mean, ─—fitted curve, ----—95% confidence band.

**Figure 4:**
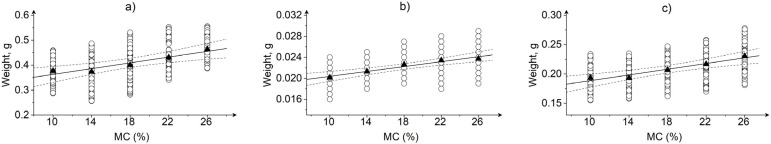
The dependencies between moisture content and weight: (**a**) maize, *m* = 0.00578 *MC* + 0.305, R^2^ = 0.914, (**b**) rice, *m* = 0.000233 *MC* + 0.0180, R^2^ = 0.953, (**c**) soybeans, *m* = 0.00243 *MC* + 0.164, R^2^ = 0.939; ○—data, ▲—mean, ─—fitted curve, ----—95% confidence band.

**Figure 5:**
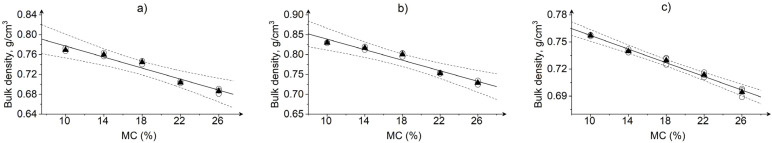
The dependencies between moisture content and bulk density: (**a**) maize, *ρ_B_* = −0.00555 *MC* + 0.833, R^2^ = 0.946, (**b**) rice, *ρ_B_* = −0.00664 *MC* + 0.905, R^2^ = 0.950, (**c**) soybeans, *ρ_B_* = −0.00379 *MC* + 0.795, R^2^ = 0.991; ○—data, ▲—mean, ─—fitted curve, ----—95% confidence band.

The results shows that the characteristic dimensions, i.e., length, width, thickness, and equivalent diameter (Figure 3), as well as the weight (Figure 4) and bulk density (Figure 5), change with MC. For the tested three grains, an increase in characteristic dimensions and weight was observed with an increase in MC. This can be confirmed by the higher than 0.88 positive Pearson correlation coefficient values and statistical significance at the level of *p* < 0.05 (Table 2). The increase in dimensions with increasing MC results were due to the specific moisture absorption behavior of grain kernels and their diffusion properties during drying [11,39]. An increase in moisture content resulted in a linear increase in grain mass (Figure 4). The weight increased by 24.18%, 19.24%, and 17.90% from the low to high MC for maize, soybeans, and rice, respectively. Changes in the water content affecting the size of the grain could be linked to swelling (during soaking) and shrinkage (during drying). The differences in dimensions with the change in MC between the types of grains can be observed in Figure 3. This is due to the difference in structure and internal composition of rice, maize, and soybeans. The increase in grain size with moisture content was confirmed by the high positive Pearson correlation coefficient values (Table 2). This relation can be described by linear or quadratic functions (Figure 3), which is consistent with the previously reported dependencies for maize [23], rice [40], and soybeans [41]. 

The change in grain size is caused by the absorption of water at the molecular level, which is greater in the case of grains containing starch [11,39,40], but smaller for grains with a vitreous structure [22]. Glassy materials are characterized by a low coefficient of expansion, diffusivity, and specific volume [22]. This explains the least susceptibility to dimensional change with increase in MC for rice, which has vitreous internal structure. On the other hand, some differences in the rate of change in dimensions in relation to MC and weight increase were observed between the grains. This is evident from the polynomial relationship between MC and length, equivalent diameter of maize kernels; thickness of rice kernels, and length of soybeans (Figure 3). In case of rice, it can be seen that the difference in kernel size at 22% and 26% MC is not as high as, for example, between kernels at 10% and 14% MC. From the relationship between weight, dimensions and MC, it can be concluded that in the case of rice grains with a moisture content of 22% and 26%, the change in weight was primarily due to the evaporation of unbound water from the grain surface, which did not cause significant changes in the grain size. A further decrease in MC below 22% resulted in a significant reduction in the dimensions of rice grains that was proportional to the weight loss. In case of maize and soybeans kernels, a faster reduction in dimensions was observed with a decrease in moisture to 18%. Below this MC value, the differences in the dimensions of grains at lower moisture content were much smaller. As a result of rapid changes in moisture content in the seed coat, additional stresses may arise, causing contraction of seed coat [42].

Research has shown that the effect of swelling as a result of water absorption or shrinkage as a result of its evaporation from the grain structure does not homogeneously affect change in size. For soybeans, the length was one of the properties affected the most by the moisture content with an observed 12.95% increase in length from the lowest average value at 14% MC to the highest at 26% MC. It is worthwhile to note that the thickness of soybeans increased only slightly (about 1.73%) with changes in MC. For rice, the thickness was the dimension influenced the most by the MC, while for maize it was width. Differences in changes in the dimensions of maize, soybeans, and rice grains may result from differences in the content of starch, proteins, and fat [43] and their ability to absorb water as well as their distribution in the internal structure, which may determine the direction of kernel enlargement caused by water absorption. As reported by Dobrzański [42] for legume seeds, including soybeans, the seed cover may experience shrinkage by up to 26% as a result of drying, and the shrinkage proportions may differ in the transverse and longitudinal directions. 

As a result of the changes in weight and grain dimensions, which are related to the grain volume and shape, a strong negative correlation between bulk density and MC was noticed (Table 2). In case of maize and rice, bulk density was the second property highly influenced by the MC, which decreased by 10.77% for maize and 12.16% for rice when the MC was increased from 10% to 26% MC (Figure 5). For soybeans, the bulk density decreased the lowest (8.27%) within the three tested grains. The increase in dimension with MC, increases contact diameter between the kernels and creation of larger void spaces resulting in an increase in porosity [44]. Differences in the dynamics of changes in bulk density between the tested types of grains are, in turn, the result of differences in the grain original size and shape characteristics, as well as their changes with MC. For soybeans, the change in dimensions with MC was not proportional in the three axes which resulted in decrease in sphericity. In contrary, the change in dimension in three axes for rice and maize was rather similar, so the sphericity increased slightly with MC. The decrease in sphericity of soybeans with the increase in MC causes a reduction in porosity. However, the soybeans’ size enlargement along the three axes has a higher influence on the porosity than change in sphericity, so the bulk density decreased. This explains the lower reduction in bulk density with the MC for soybeans compared with maize and rice kernels.

### 3.2. Effect of Moisture Content on the Shape of Grain Kernels

**Figure 6:**
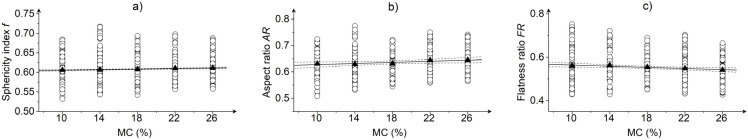
The relationship between moisture content and selected shape characteristics for maize: (**a**) sphericity, *f* = 0.000353 *MC* + 0.602, R^2^ = 0.922, (**b**) aspect ratio, *AR* = 0.00105 *MC* + 0.617, R^2^ = 0.781, (**c**) flatness ratio, *FR* = −0.00132 *MC* + 0.577, R^2^ = 0.873; ○—data, ▲—mean, ─—fitted curve, ----—95% confidence band.

**Figure 7:**
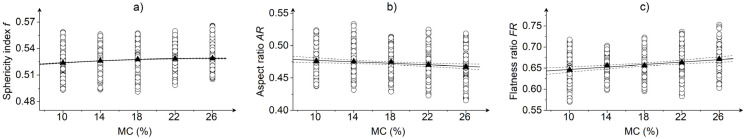
The relationship between moisture content and selected shape characteristics for rice: (**a**) sphericity, *f* = −0.000021 *MC* + 0.515, R^2^ = 0.999, (**b**) aspect ratio, *AR* = −0.00058 *MC* + 0.483, R^2^ = 0.887, (**c**) flatness ratio, *FR* = 0.00147 *MC* + 0.632, R^2^ = 0.945; ○—data, ▲—mean, ─—fitted curve, ----—95% confidence band.

**Figure 8:**
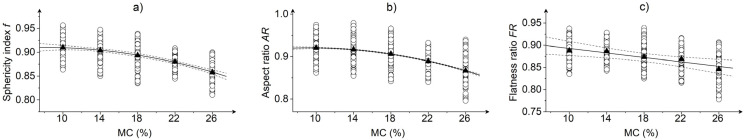
The relationship between moisture content and selected shape characteristics for soybeans: (**a**) sphericity, *f* = −0.000158 *MC*^2^ + 0.00249 *MC* + 0.900, R^2^ = 0.998, (**b**) aspect ratio, *AR* = −0.000193 *MC*^2^ + 0.00357 *MC* + 0.904, R^2^ = 0.999, (**c**) flatness ratio, *FR* = −0.00254 *MC* + 0.918, R^2^ = 0.891; ○—data, ▲—mean, ─—fitted curve, ----—95% confidence band.

Due to the changes in the dimensions of the grains with the change in MC, as previously discussed, the changes in grain shape were also noticeable (Figure 6, Figure 7 and Figure 8). The soybean kernels have the shape closer to spherical among the studied grains, as evidenced by sphericity index, flatness, and aspect ratio values that are close to 1 (Figure 8). As well known, the rice kernels have the most elongated shape (Figure 7).

The sphericity, aspect ratio, and flatness ratio were negatively correlated with MC for soybeans (Table 3) and decreased by about 4.7–5.8% from 10% to 26% MC. As was shown in the previous section, the thickness of soybeans changed only slightly with MC, while the length and width increased significantly, resulting in the shape of soybeans to become more elongated and less spherical. Thus, at higher MC, the rolling ability of soybeans will decrease, and the interparticle contact will change, and the pattern of filling a given volume. For soybean kernels, the relationship between MC and flatness ratio had a very good linear fit (R^2^ = 0.891) (Figure 8c). This resulted from the linear increase in grain thickness and width with MC. Sphericity and aspect ratio, as the resultant of nonlinear change of length with MC, take the form of a quadratic function (R^2^ = 0.998 and R^2^ = 0.999 respectively, Figure 8a,b).

Contrary to soybeans, the shape of maize and rice grains changed only slightly when the MC increased. A positive correlation was noticed between MC and sphericity of these kernels (Table 3), however, the increase was only about 1% from the lowest to the highest MC level. As discussed earlier, water absorption caused an almost uniform increase in the dimensions of rice and maize grains along the three axes, hence slight changes in sphericity (Figure 6a, Figure 7a). As a consequence, the changes in aspect ratio and flatness ratio of these grains were minimal (Figure 6b, Figure 7b). The aspect ratio of rice kernels decreased with the MC (negatively correlated, Table 3) by about 1.90% at 26% MC compared to the kernels at 10% MC. This was caused by a slightly greater expansion in the longitudinal direction than in the transverse direction. The greater increase in kernel thickness in relation to its width resulted in an increase in flatness ratio by 3.99%. For maize, however, the flatness ratio decreased by about 3.94% for kernels at 26% MC compared with grains at 14% MC, and the aspect ratio increased by about 2.42%. The relationship between MC and sphericity, aspect ratio, and flatness ratio for rice and maize kernels were linear (R^2^ > 0.781 for all fitted relationships, Figure 6 and Figure 7). 

The difference in changes in shape parameters with the MC of different types of grains could be due to their variation in internal structure and composition. As observed, the change in the shape of the grain depends on the changes in dimensions due to changes in the moisture content. Slight changes in sphericity, aspect ratio, and flatness ratio for rice and maize indicate that for these grains the change in dimensions due to water content changes occurred almost proportionally in three axes, with only a slightly larger change in one of the dimensions in relation to the others, hence the observed 2–3% difference in shape characteristics between wet (26% MC) and dry (10% MC) grains. 

Rice and maize consist mainly of starchy endosperm with varying degrees of packing. Maize additionally has an outer pericarp layer that is different than rice kernels. In soybeans, the main components are an embryo and two cotyledons surrounded by a seed coat with hilum that holds the seed to the pod. The presence of hilum may be the impediment for the seed coat deformation, forcing the soybean kernel to expand less along its thickness. The arrangement of cotyledons and their separation in the plane, determined by the length and width of the grain, also may have an influence on the non-uniform change in dimensions in three axes, and thus change the shape. In the case of maize, the loose structure of the starchy endosperm allows the cells to expand while absorbing water, which may result in changes in starch arrangements [12], thus swelling occurs in all directions. The shape of soybeans changed the most within the tested grains, which shows that it becomes more flexible with the increase of MC than rice and maize kernels.

### 3.3. Effect of Moisture Content and Loading Rate on the Mechanical Behavior of Grain Kernels during Compression

**Figure 9:**
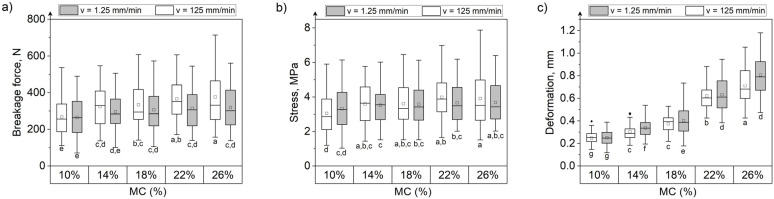
The breakage force (**a**), stress (**b**), and deformation (**c**) during maize grain compression at five levels of moisture content and two loading rates. Letters below the box charts are the interaction grouping letters from the post hoc Fisher least significant difference means comparison test. Means that do not share a letter are significantly different. □—mean, ──—median, ◆—outliers.

**Figure 10:**
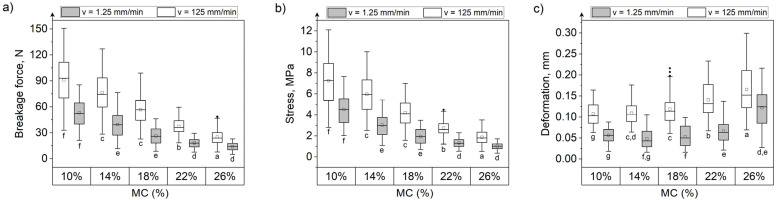
The breakage force (**a**), stress (**b**), and deformation (**c**) during rice grain compression for five levels of moisture content and two loading rates. Letters below the box charts are the interaction grouping letters from the post hoc Fisher least significant difference means comparison test. Means that do not share a letter are significantly different. □—mean, ──—median, ◆—outliers.

**Figure 11:**
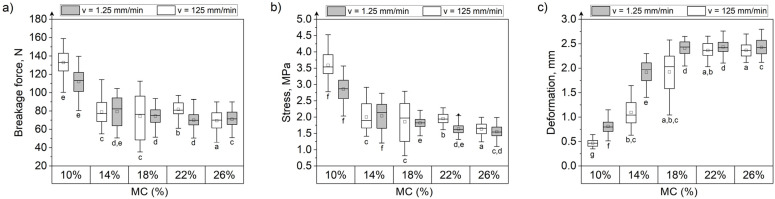
The breakage force (**a**), stress (**b**), and deformation (**c**) during soybean compression for five levels of moisture content and two loading rates. Letters below the box charts are the interaction grouping letters from the post hoc Fisher least significant difference means comparison test. Means that do not share a letter are significantly different. □—mean, ──—median, ◆—outliers.

MC had the greatest influence on the deformation of all tested grain kernels at both the loading speeds (Figure 9, Figure 10 and Figure 11). Deformation increased approximately 3.2 times, 2.53 times, and 3 times, respectively, for maize, rice, and soybeans at a MC of 26% compared with kernels at 10% MC when compressed at 1.25 mm/min. At 125 mm/min, the increase in deformation for grains with 26% moisture compared with grains at 10% moisture was 2.88 times for maize, 1.5 times for rice, and 2.92 times for soybeans. The greatest difference in forces and stresses between grains at 10% and 26% MC was recorded for rice grains (Figure 10). Forces and stresses decreased by approximately 74% (72% when compressed at a speed of 125 mm/min) and 78% (74% at 125 mm/min loading rate), respectively. The lowest impact of MC on the change in forces and stresses was noted for maize kernels (Figure 9). The breaking forces for maize kernels at 26% moisture increased by 20.3% (40.3% at 125 mm/min loading rate), and stresses by 11% (28.3% at 125 mm/min loading rate) compared to 10% moisture grains.

**Figure 12:**
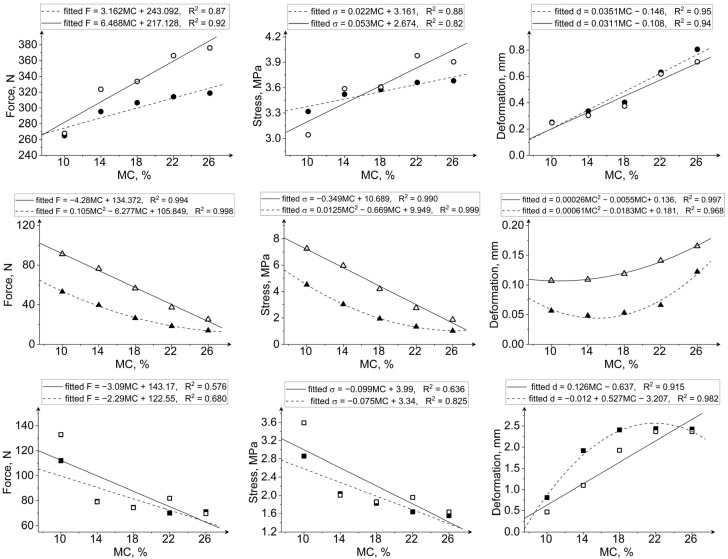
The dependencies between moisture content and selected mechanical properties for maize (circle), rice (triangle), and soybean (square) kernels at two loading rates: 1.25 mm/min (black) and 125 mm/min (white).

At the two loading rates, the changes in force, displacement, and stress with the MC follow the same relationship. The breakage forces increased with the increase in MC for maize (positive correlation, r = 0.930, Table 4), while in the case of rice and soybeans the breakage forces decreased (negative correlation, Table 4). Similarly, the stress values for maize increased with MC (positive correlation, Table 4), and an inverse relationship was observed for rice and soybeans (negative correlation, Table 4). The deformation of kernels increased with moisture content for all three types of grains tested. The relationship between MC and destructive force, deformation, and stress can be described with a linear or quadratic relationship, as shown in Figure 12.

Based on the presented results, it can be observed that the grains with low MC had lower deformations during compression, and their mechanical behavior was similar to brittle materials. However, kernels with high MC were softer and showed greater plastic deformation ability. All three tested grains became softer from water absorption. However, maize kernels, having a fibrous outer layer, were elastic and resistant to damage at high MC. This agrees with the studies that reported lower breakage rates for maize grains at higher MC within the range of 8 to 25% [45,46]. Zdunek et al. [47]) stated that the high turgor could be responsible for higher resistance of materials to loading, since it may lead to high cell wall tension. Although soybeans have a seed coat, their load susceptibility decreases, as is the case with rice. The seed coat of soybeans is mainly composed of protein, and, as indicated by Li et al. [48], soaking causes the seed coat to become increasingly less packed with larger gaps between cells due to the release of proteins. It was previously reported that the cell packing as well as the occurrence of biochemical reactions can affect the mechanical properties [49]. Moreover, soybeans undergo severe deformation at higher MC, which could be due to the increase in stress on the seed coat. Dobrzański [42] stated that the fat content changes with the MC and this could be responsible for the high deformability of wet soybeans. Qiao et al. [12] showed that the protein content is responsible for grain hardness and elastic properties, while the starch is responsible for viscosity, and that during water absorption, the pores between starch granules increase so viscosity and deformation are higher, which was observed for rice and maize. The ductile characteristic of soybeans is related to the oil content [50]. Higher oil content increases the breakage energy, due to an increase in ductility [50]. Moreover, it was found that during soaking, some changes in the crude protein, crude ash, and starch content may occur, resulting in structure modification, which may influence the ability to bind water and deform their shape [43]. 

It has been reported that interaction between carbohydrates and proteins [12], structural composition, density, cell adhesion, and turgor pressure of cells [47] are the important factors that affect the mechanical behavior of cereal grains. Other factors include the ratio of horny to floury endospherm [23], size, shape, temperature, and strain rate [12,30,49,51]. It has also been proven that the horny endosperm, germ, and floury endosperm show different mechanical properties, with the horny endosperm having a lower deformation and able to sustain higher forces than the floury endosperm [27]. Furthermore, Dong et al. [52] has shown that the number of closed pores, surface of closed pores, and closed porosity percent affect the breakage resistance.

For rice kernels, a significant difference based on the result of the two-way ANOVA test and the Fisher LSD test between forces causing breakage, stress, and deformation at two loading rates was observed. For rice, the force, stress, and deformation were higher at a loading rate of 125 mm/min than at the low loading rate of 1.25 mm/min. The increase in stresses in rice subjected to loading at a higher rate could be due to fast crack propagation, making it impossible to differentiate the force causing breakage initiation from the force leading to grain rupture. 

In the case of maize and soybeans, the influence of the loading rate on the breakage behavior is not obvious. For maize, slightly higher fracture forces were recorded during the compression test at a speed of 125 mm/min. However, statistically significant differences between the mean values for the two compression speeds were obtained only for grains at a moisture content of 22% and 26% (Figure 9). There were no statistically significant differences between the mean stresses for the two different loading speeds. The deformations obtained during compression at a speed of 125 mm/min turned out to be slightly lower than during compression at a speed of 1.25 mm/min, however, statistically significant differences occurred between the average deformations for grains with two moisture levels of 14% and 26%. 

For soybeans, statistically significant differences between the average breaking forces and the stresses (higher at the speed of 125 mm/min) at the two loading speeds were noticed only for grains at 10% and 22% moisture content (Figure 11). However, the influence of the loading speed on the forces and stresses is not clear. The mean deformations of soybeans during the compression test at a speed of 125 mm/min were lower than those at the low loading rate. 

The increase in force and stress during loading at a higher rate is a result of higher internal friction between cells inside the cereal grain kernels. This could be also the reason for lower deformation at the higher loading rate, as the internal pressure increases due to an increase in cell packing, so the failure starts quicker. Moreover, in contrast to rice and soybeans, maize kernels have a seed coat that affects the pattern of deformation during compression at different speeds. The presence of a seed coat may provide an additional internal stress during compression. When kernels experience transverse deformation, cells move perpendicular to the direction of loading and the seed coat applies additional pressure within the kernels [42]. For rice kernels, without a seed coat, the force is applied directly on the endosperm. Hebda and Frączek [53] found that the seed outer layer is more flexible, transfers load during compression, and is responsible for maintaining (bonding) within the internal structure. Their study claimed that the kernels can be treated as thin-walled vessels filled with a material of a certain viscosity. So, due to the increased water content, the internal pressure in grains with a seed coat will increase, causing additional stress. This could explain the higher stresses and forces with lower deformation observed for maize and soybeans during compression at the high loading rate. Moreover, Hebda and Frączek [53] stated that the thickness of the seed coat affects the elasticity of the grains and their ability to deform. The presence of the seed coat will have a strong influence on the kernel deformation, especially at high water content. Wet maize kernels can sustain higher force, stress, and deformation, as they have a fibrous outer layer with elastic behavior. In the wet state, the outer layer is more resistant to cell movement and internal pressure induced in the softer endosperm due to increased deformability. Rice does not have a seed coat, so, at high moisture conditions it undergoes higher deformation due to softening caused by the water absorption, but can sustain the lower forces and stress in dry conditions. In the case of soybeans, with increasing moisture content, very high deformations were observed because of the softening of the internal structure and the high elasticity of the seed coat. 

A literature review shows different observations for changes in forces, stresses, and deformations for the point of breakage initiation with the loading rate. Tavakoli et al. [28] reported lower rupture forces during compression of barley at a 10 mm/min loading rate than at a 5 mm/min loading rate. Similar results were reported for wheat [54] and rice [30]. The forces were lower during compression of cumin seed at a loading rate of 5 mm/min than at a loading rate of 2 mm/min [29]. Su et al. [51] reported a decrease in forces with loading rate for pyramidal and rectangularly cut maize kernels during compression along the X, Y, and Z axes, and an increase in force with loading rate for round kernels along the Z axis. Similar to the results presented in this study for rice, Zhao et al. [55] showed that the deformation during compression of oat kernels had an increasing trend with the increase in loading rate in the range of 0.02–0.10 mm/s, and the forces and stresses initially increased and then decreased with an increase in loading rate. On the contrary, Uguru et al. [56] reported that the deformation during compression of groundnut decreased with the increase in loading rate (15, 20, and 25 mm/min), which was also observed in our study for maize and soybeans. 

The different observations of changes in the forces, stresses, and deformations at bioyield or rupture point with the changes in loading rate indicate that further research is needed to establish reliable dependencies, especially in a wider range of loading rates, while most of the studies report the results for narrow ranges of loading rate variability. 

### 3.4. Consequences of Variable Grain Moisture Content on the Grain Processing and Machine Design

Variability of physical properties such as grain size, shape, weight, and bulk density is of great importance in the design of machines and processing processes. The values of grain dimensions and shapes are necessary for calculating and optimizing the sieves and sieves opening design, which determine sieving efficiency. The significant differences in weight, shape, and dimensions at low and high MC indicate that it may be necessary to use different sets of sieves for the classification and separation processes for grains. Changes in the rolling ability of materials and changes in contact points with the surface that characterizes grain flowability and behavior during handling processes should also be considered when calculating the optimal angles for conveying and feeding equipment. The changes in particle sizes and shapes are responsible for changes in bulk density, which will cause changes in the pressure on the storage equipment walls and transmission of loads [5]. As indicated by Horabik and Rusinek [5], the pressure ratio, bulk density, and friction coefficient are the most important parameters that should be taken into account when determining loads in containers, silos, and other storage structures. Bulk density decreases with the increase of MC, as shown in this study and other studies [1]. Dale and Robinson [57] showed that with an increase in MC by 4% w.b., the lateral and vertical loads increased by 6 and 4 times, respectively. The results from this research show that in storage equipment design, the variability of bulk density in MC ranges from 10% to 26%, with values of about 10.8%, 8.3%, and 12.2% for maize, rice, and soybean kernels, respectively, should be considered. The mentioned changes in pressure and loads induced by the moisture content variability will have an influence on the storage equipment geometry (height, width, diameter, etc.) and calculated wall thickness, and will affect the choice of the material with appropriate strength. 

**Figure 13:**
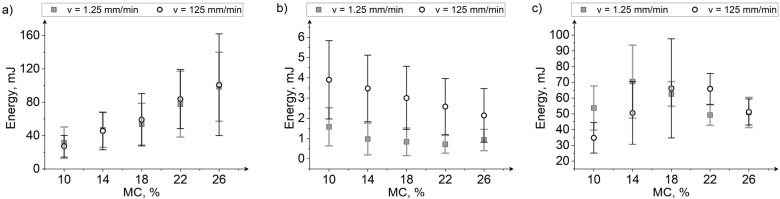
The dependence between moisture content and energy needed for single grain compression; (**a**) maize, (**b**) rice, (**c**) soybeans.

With changes in mechanical properties influenced by moisture, the energy needed to break the grain also changes, which is of great importance in industrial processes, especially in the context of energy consumption during grinding, component extraction, and flour preparation [3,12,28,58]. In this study, the energy for grain breakage was calculated as the area under the force–deformation curve. The energy values are then dependent on the values of breakage forces and deformations to damage, as well as on the slope of the force–deformation curve. Thus, the trends in energy changes with MC will be similar to the case of changes in forces and deformations. Figure 13 shows the energy range required to initiate cracking in maize, rice, and soybean kernels at different moisture and loading rates. The results of ANOVA show that the moisture content has a significant influence on the energy needed for breakage initiation in maize, rice, and soybeans. Significant differences (based on the Fisher LSD post hoc test) between the means of grains with different moisture contents were observed (Table 5). 

The breakage energy of maize kernels increased with an increase in MC (Figure 13a). The increase in MC resulted in grains becoming more deformable and withstanding greater loads than dry grains (Figure 9a). Maize kernels at higher MC will therefore be more resistant to damage caused by external loads. However, the load-bearing capacity of maize will depend heavily on its variety and the content of horny and floury endosperm, which is unique to each grain variety [23]. Wang and Wang [27] indicated that the soft, floury endosperm is characterized by lower resistance to loads and greater deformation than the horny endosperm. Thus, the predominance of one type of endosperm over the other will determine the mechanical properties such as force, deformation, and energy needed for failure. An increase in MC decreased the yield point for rice kernels (Figure 13b). The absorption of water by the grain kernels resulted in a significant reduction of forces and strength (Figure 10a,b), despite the observed increase in deformation. The energy and mechanical properties are closely related to the glassy structure of the rice, in which the starch cells are tightly packed. Under the influence of water, the intercellular distance changes (the grain volume increases), causing the mobility of starch chains, thus increasing the plasticity of the grain and lowering its hardness [12,22]. Rice grains with low moisture are characterized by high hardness, which decreases with increasing MC. On the other hand, the reduction of hardness contributes to the reduction of breakage force and energy during compression [59]. The obtained results indicate that rice grains will be most susceptible to damage at high MC values and even small loads of a few kilograms during processing operations will damage them. For soybeans, it was observed that with the increase in MC, the energy first increased and then decreased (Figure 13c). The highest energy values were observed for soybeans at an MC of 14% and a loading rate of 1.25 mm/min (73.26 mJ), and at 125 mm/min loading rate for soybeans with MC 18% (66.16 mJ). The observed decrease in energy for soybean grains at high MC is due to a slight increase in the deformation with the MC and higher differences in forces. At 10% MC, the soybean kernels were characterized by higher hardness than the wet grains, as evidenced by higher force values and smaller displacements for grains at lower MC. The highest energy values for 14–18% MC may prove that the soybeans grains are visco-elastic in this MC range, and above 18% MC, plastic properties are dominant. In the range of 22–26% MC, no increase in forces and deformations for soybeans was observed, which may be related to the seed coat reaching the maximum tensile strength. This results in no further deformation to breakage of soybeans with increasing MC, as well as only slight changes in breakage forces, as the less strong seed coat will crack first, although the cotyledons will be able to deform plastically further. The relation between soybean breakage energy and MC strongly depended on changes in mechanical properties (quadratic relationship for both loading rates, Figure 12) with changes in water content. As indicated by Dobrzański [42], with the change in moisture content in soybean kernels, the share of fat in the total weight of the grain changes and shows plastic properties with high water content. This suggests that soybeans will be damaged more likely at low MC values, while in the high MC (above 18%) the damage will appear on the seed coat but not necessarily in the cotyledons.

It was also found that the influence of loading rate was significant only for rice and soybeans (Table 5). A detailed comparison between means considering the interaction effect of MC and loading rate is presented in Table 6. The lack of statistically significant differences in the breakage energy for maize compressed at two loading rates results from the fact that both the forces and deformations for the two loading rates were at a similar level in the tested MC ranges. An increase in loading rate caused the energy needed to reach yield point during rice compression to increase. The observed increase of forces and deformations when loading at higher velocity lead to higher breakage energy values in rice grains. For soybeans, statistically significant differences in energy between the two loading rates were noted for beans at 10%, 14%, and 22% MC. The increase in energy in the MC range of 10% to 14% with decreasing load rate results from greater deformations obtained at a lower deformation rate. The occurrence of a crack at lower deformations at a loading rate of 125 mm/min may be due to cell accumulation as a result of an increase in the deformation speed. At 18% and 22% MC at 125 mm/min speed, higher values of destructive energy were obtained than at the speed of 1.25 mm/min (at 18% the difference was statistically insignificant), which may be due to the plasticization of soybeans at higher MC and then increased resistance of moist grains during rapid deformation. At 26% MC, the breakage energy values of soybean kernels at both speeds were comparable (Figure 13), as were the values of forces and deformation (Figure 12).

The presented results suggest that the value of the breakage energy will depend on the properties of the materials and their behavior under loads, and more precisely on the elastic and plastic deformations during loading. Moisture content changes the proportion of elastic and plastic deformations in the total grain deformation, which seems to have an impact on changes in energy values during loading at different speeds. 

The presented results are of great importance for harvesting, conveying, storage, and comminution equipment design. On one hand, the preferred moisture content ranges ensuring the low rate of damage during grain harvesting, storage, and transporting operations (or high damage rate for comminution equipment) can be selected [60]. On the other hand, presented ranges of kernel mechanical parameters for various MC and loading rates can support the optimization of loads acting on the grains in processing equipment. The load influencing the kernel in agricultural machines is a result of elements geometry and operational parameters such as velocity, rotational speed, and contact frequency [61]. The knowledge about the mechanical responses of grain subjected to loads is then crucial to control and optimize the design and operational parameters of processing machines. The applications of forces and energies of kernel breakage in the comminution equipment design can lead to the increase in their efficiency and power consumption reduction. 

The strong influence of moisture content on breakage characteristics, described in this study, was also evidenced in the research on the comminution equipment reported by other researchers. During hammermilling of maize, the increase in specific comminution energy and decrease in throughput and breakage rate were observed with an increase in MC [32]. For rice grinding on a five-disc mill, an increase in specific energy and size reduction rate was observed with an increase in MC, while the throughput decreased with an increase in MC [62]. Although during single rice kernel compression a decrease in the energy required for grinding was observed, in the case of grinding in a mill, an increase in energy was observed with an increase in MC. This may be caused by a greater increase in energy due to the friction of rice grains between each other and between the grinding elements compared with the increase in energy on the effect of changes in MC. 

## 4. Conclusions

This paper presents an analysis on the influence of moisture content on changes in selected size and shape characteristics as well as the mechanical properties of maize, rice, and soybean kernels. It was found that an increase in moisture content causes an increase in grain size and a change in their shape. For rice and maize, the sphericity of grains increased with the increase in moisture, while the opposite relationship was observed for soybeans. The increase in grain moisture content also changed the forces, stresses, and strains causing grain cracking. A significant increase in deformation was observed with increasing moisture content for the three tested grains. In the case of maize, there was also an increase in forces and stresses causing fracture with increasing moisture content, while for rice and soybean grains an opposite relationship was observed. The results indicate a strong relationship between the moisture content and mechanical properties. It should also be noted that an increase in the moisture content of the grains contributes to the change in the nature of the crack propagation, from more brittle at low moisture to a more plastic behavior at higher moisture content. In the case of rice, it was found that increasing the compression speed increased the forces, stresses, and strains during compression. For maize and rice, these relationships were not so obvious, although a slight increase in forces and stresses as well as a reduction in strain was observed. The relationship between moisture content and selected physical and mechanical properties can be described using linear or quadratic functions. 

The results indicate that changes in physical and mechanical properties should be taken into account in the process of designing machines, devices, and processes of cereal grains, especially the changes in bulk density (approximately 20% from the lowest to the highest moisture content) and forces causing fracture (approximately 75% decrease in forces for high moisture kernels for rice grains, about 43% decrease for soybeans, and about 20% increase for maize kernels). The significant influence of moisture content on the energy inducing the breakage was observed for all three tested grains. The increase in the loading rate causes an increase in energy in the case of rice and a decrease for soybeans. The presented energy values and the relationships with MC constitute an important indicator of the grain’s mechanical resistance to loads during mechanical operations and, considering the design process, can help to reduce material losses due to damage.

## Figures and Tables

**Table 1:** Moisture content of cereal grains after conditioning to predetermined levels.

Moisture Content, % w.b.			
	Maize	Rice	Soybeans
Level 1: 10%	10.02	9.98	9.76
Level 2: 14%	13.95	13.96	14.10
Level 3: 18%	18.02	17.91	17.92
Level 4: 22%	22.09	21.80	21.94
Level 5: 26%	26.40	25.71	26.19

**Table 2:** Results of correlation analysis between moisture content and physical properties of grain kernels.

		Thickness	Length	Width	Equivalent Diameter	Weight	Bulk Density
Maize	Pearson Corr.	0.961 *	0.968 *	0.946 *	0.939 *	0.956 *	−0.972 *
	*p*-value	0.009	0.007	0.015	0.018	0.011	0.005
Rice	Pearson Corr.	0.956 *	0.963 *	0.892 *	0.937 *	0.976 *	−0.975 *
	*p*-value	0.011	0.008	0.042	0.019	0.004	0.005
Soybeans	Pearson Corr.	0.889 *	0.958 *	0.935 *	0.899 *	0.969 *	−0.996 *
	*p*-value	0.045	0.010	0.020	0.038	0.007	0.000

A 2-tailed test of significance is used, *: Correlation is significant at the 0.05 level.

**Table 3:** Results of correlation analysis between moisture content and selected shape characteristics (based on average values).

		Sphericity	Aspect Ratio	Flatness Ratio
Maize	Pearson Corr.	0.960 *	0.884 *	−0.934 *
	*p*-value	0.009	0.047	0.020
Rice	Pearson Corr.	0.953 *	−0.942 *	0.972 *
	*p*-value	0.012	0.017	0.006
Soybeans	Pearson Corr.	−0.973 *	−0.965 *	−0.944 *
	*p*-value	0.005	0.008	0.016

A 2-tailed test of significance is used, *: correlation is significant at the 0.05 level.

**Table 4:** Results of correlation analysis between moisture content and mechanical characteristics (based on average values).

		Loading Rate 1.25 mm/min			Loading Rate 125 mm/min		
		Breakage Force	Breakage Stress	Deformation	Breakage Force	Breakage Stress	Deformation
Maize	Pearson Corr.	0.930 *	0.940 *	0.974 *	0.959 *	0.907 *	0.968 *
	*p*-value	0.022	0.017	0.005	0.010	0.033	0.006
Rice	Pearson Corr.	−0.980 *	−0.965 *	0.777	−0.997 *	−0.995 *	0.949 *
	*p*-value	0.003	0.008	0.122	1.81 × 10^−4^	4.19 × 10^−4^	0.014
Soybeans	Pearson Corr.	−0.825 ^#^	−0.908 *	0.847 ^#^	−0.759	−0.797	0.957 *
	*p*-value	0.086	0.033	0.070	0.137	0.106	0.011

Results based on 2-tailed test of significance, *: correlation is significant at 0.05 level, ^#^: correlation is significant at 0.1 level.

**Table 5:** The means comparison of energy needed to induce breakage at different moisture contents and loading rates.

	Energy (mJ)		
	Maize	Rice	Soybeans
Loading rate			
1.25	61.14(a)	1.01(b)	58.02(a)
125	62.36(a)	3.02(a)	53.88(b)
MC			
10%	29.53(e)	2.69(a)	43.80(e)
14%	46.25(d)	2.21(b)	61.86(b)
18%	56.39(c)	1.87(c)	65.17(a)
22%	80.62(b)	1.61(d)	57.36(c)
26%	99.76(a)	1.53(d)	51.40(d)

(): grouping letters of the post hoc Fisher least significant difference means comparison test; means that do not share a letter are significantly different.

**Table 6:** The means comparison of energy needed to induce the breakage for interactions effect of moisture content and loading rates.

	Energy (mJ)					
	Maize		Rice		Soybeans	
Loading Rate	1.25 mm/min	125 mm/min	1.25 mm/min	125 mm/min	1.25 mm/min	125 mm/min
MC						
10%	31.57(e)	27.29(e)	1.58(f)	3.90(a)	53.01(c)	34.79(e)
14%	46.96(d)	45.54(d)	0.98(g)	3.48(b)	73.26(a)	50.58(c,d)
18%	53.68(c,d)	59.10(c)	0.72(g)	3.00(c)	64.07(b)	66.16(b)
22%	77.74(b)	83.82(b)	0.84(g)	2.58(d)	48.19(d)	65.87(b)
26%	98.62(a)	100.98(a)	0.93(g)	2.14(e)	51.57(c,d)	51.22(c,d)

(): interaction grouping letters of the post hoc Fisher least significant difference means comparison test; means that do not share a letter are significantly different.

## Data Availability

The data presented in this study are openly available in [ZENODO] at [10.5281/zenodo.6390910].
